# Outcomes of Radiotherapy for WHO Grade 2 Meningioma: Survival, Local Control, and Prognostic Factors

**DOI:** 10.3390/medicina62081523

**Published:** 2026-08-07

**Authors:** Rahşan Habiboğlu, İlknur F. Kayalı, Gonca Altınışık İnan, İpek Pınar Aral, Sedef Gökhan Açıkgöz, İrem Sarıcanbaz, Binnur Dadak, Merve Ertürk, Yılmaz Tezcan

**Affiliations:** 1 Department of Radiation Oncology, Ankara Bilkent City Hospital, 06800 Ankara, Türkiye; habiboglu@gmail.com (R.H.); goncaaltinisikinan@gmail.com (G.A.İ.); ipekpt@hotmail.com (İ.P.A.); drsedeff@gmail.com (S.G.A.); iremsaricanbaz@gmail.com (İ.S.); tuncerbinnur@gmail.com (B.D.); drmerveerturk@gmail.com (M.E.); yilmaztezcan@yahoo.com (Y.T.); 2 Department of Radiation Oncology, Ankara Yıldırım Beyazıt University Faculty of Medicine, 06800 Ankara, Türkiye

**Keywords:** atypical meningioma, WHO grade 2 meningioma, postoperative radiotherapy, overall survival, local control, prognostic factors

## Abstract

*Background and Objectives*: The optimal role of postoperative radiotherapy and the prognostic factors influencing outcomes in patients with WHO grade 2 meningioma remain controversial. This study evaluated survival outcomes, local control, treatment-related toxicity, and prognostic factors in patients treated with postoperative radiotherapy. *Materials and Methods*: This retrospective single-center study included 50 consecutive adult patients with histologically confirmed WHO grade 2 meningioma who underwent surgical resection followed by radiotherapy between 2013 and 2024. Overall survival (OS) and local control (LC) were estimated using the Kaplan–Meier method, and median follow-up was calculated using the reverse Kaplan–Meier method. Univariable and multivariable Cox proportional hazards regression analyses were performed to identify prognostic factors associated with OS, while univariable analyses were performed for LC. *Results*: The reverse Kaplan–Meier median follow-up was 20.9 months (95% CI, 13.2–34.8). During follow-up, 10 patients died and 7 developed local recurrence. Median OS was 59.3 months (95% CI, 49.9 months–not estimable). The estimated 2- and 5-year OS rates were 85.0% and 44.8%, respectively, while the corresponding LC rates were both 76.2%. Increasing age and larger tumor size were independently associated with worse OS. All deaths and all local recurrences occurred in patients with postoperative residual disease. Acute adverse events were limited to Grade 1–2 toxicity, and no Grade ≥3 acute or late toxicities were observed. *Conclusions*: Postoperative radiotherapy achieved favorable local control with an acceptable safety profile in patients with WHO grade 2 meningioma. Postoperative residual disease was strongly associated with adverse clinical outcomes, while increasing age and larger tumor size were independently associated with worse overall survival. These findings support the importance of maximal safe resection, careful postoperative risk stratification, and individualized multidisciplinary treatment planning.

## 1. Introduction

Meningiomas are the most common primary intracranial tumors in adults, accounting for approximately one-third of all central nervous system neoplasms. Although the majority are classified as WHO grade 1 and generally exhibit an indolent clinical course, atypical meningiomas (WHO grade 2) represent a biologically more aggressive subgroup associated with increased recurrence rates, shorter progression-free survival, and a greater likelihood of requiring multimodality treatment. The 2021 World Health Organization classification further refined the diagnostic criteria for meningiomas and highlighted the growing importance of histopathological and molecular characteristics in predicting clinical behavior and guiding treatment decisions [1,2].

Maximal safe surgical resection remains the cornerstone of treatment for atypical meningiomas. However, surgery alone is frequently insufficient to achieve durable disease control. Reported recurrence rates after gross total resection range from approximately 20% to 50%, while patients with subtotal resection or residual disease experience substantially higher rates of progression [3,4,5,6]. Consequently, postoperative radiotherapy has become an integral component of treatment for many patients with WHO grade 2 meningiomas, particularly in the presence of residual tumor, recurrent disease, or other high-risk features [2].

Despite its widespread use, the optimal role of radiotherapy in atypical meningioma remains controversial. Several retrospective studies have demonstrated improved local control and progression-free survival following adjuvant radiotherapy, especially after subtotal resection or in patients with residual disease [7,8,9]. However, the benefit of routine postoperative radiotherapy following gross total resection remains less certain. Multiple institutional series have reported conflicting results, and considerable variation persists in treatment recommendations across centers [7,8,9,10]. To address this uncertainty, several meta-analyses have evaluated the impact of adjuvant radiotherapy after surgery and generally report improved disease control, although its effect on overall survival remains less clearly established [11,12,13,14].

Prospective studies have strengthened the evidence supporting postoperative radiotherapy in selected patients. The NRG Oncology RTOG 0539 study demonstrated excellent local control and progression-free survival in intermediate-risk meningiomas treated with adjuvant radiotherapy, while the EORTC 22042-26042 phase II trial reported favorable outcomes after high-dose postoperative radiotherapy for atypical and anaplastic meningiomas [15,16]. At the same time, the ongoing ROAM/EORTC 1308 randomized trial is expected to provide higher-level evidence regarding the role of adjuvant radiotherapy following gross total resection of atypical meningiomas [17].

In recent years, stereotactic radiosurgery and stereotactic radiotherapy have emerged as attractive treatment options for selected patients with limited residual or recurrent disease. Nevertheless, local control remains suboptimal in a subset of high-risk patients, and the precise role of stereotactic approaches within the broader management paradigm of atypical meningioma continues to evolve [18].

Beyond treatment selection, the identification of reliable prognostic factors remains an important clinical challenge. Previous studies have suggested associations between survival outcomes and factors such as extent of resection, residual disease, tumor size, proliferative activity, and performance status [5,19,20,21,22,23]. However, reported findings have been heterogeneous, reflecting differences in patient populations, treatment strategies, pathological classification systems, and radiotherapy practices. As a result, prognostic stratification and treatment individualization remain areas of ongoing investigation.

Given the persistent uncertainty regarding both the optimal use of radiotherapy and the prognostic determinants of outcome in atypical meningioma, we retrospectively evaluated a homogeneous cohort of patients with histologically confirmed atypical meningioma (WHO grade 2) treated with radiotherapy at our institution. The primary objective of this study was to evaluate overall survival and local control after radiotherapy in patients with WHO grade 2 meningioma. A secondary objective was to identify independent clinical and treatment-related prognostic factors associated with overall survival.

## 2. Materials and Methods

### 2.1. Study Design

This retrospective single-center study included consecutive adult patients with histopathologically confirmed atypical meningioma (WHO grade 2) who underwent surgical resection followed by radiotherapy between January 2013 and December 2024. The study was approved by the local Ethics Committee (approval date: 6 November 2024; approval number: TABED1-24-709) and was conducted in accordance with the Declaration of Helsinki.

### 2.2. Patient Characteristics

Eligible patients were aged ≥18 years and had available clinicopathological, radiotherapy, and follow-up data. Patients with WHO grade 1 or grade 3 meningiomas, patients without histopathological confirmation, patients who did not receive radiotherapy, and patients with incomplete clinical, radiological, or treatment-related data were excluded.

A total of 50 patients met the eligibility criteria and were included in the final analysis. Recorded variables included age, sex, ECOG performance status, tumor size, tumor location, postoperative residual disease status, Ki-67 index, progesterone receptor expression, radiotherapy indication, radiotherapy technique, treatment response, recurrence status, and survival outcomes.

A summary of the baseline patient and tumor characteristics is presented in Table 1.

### 2.3. Treatment Characteristics

Radiotherapy was administered as adjuvant treatment following primary surgery, adjuvant treatment after surgery for recurrent disease, or salvage treatment for recurrent tumors. Primary adjuvant radiotherapy was defined as radiotherapy delivered after the initial surgical resection of a WHO grade 2 meningioma. Adjuvant radiotherapy after recurrence surgery was defined as radiotherapy administered following resection of recurrent disease. Salvage radiotherapy was defined as radiotherapy delivered for recurrent or residual disease without additional surgical intervention.

All patients underwent contrast-enhanced postoperative magnetic resonance imaging (MRI) approximately one month after surgery to assess the presence of residual disease. Postoperative residual disease status was determined based on these MRI findings and was incorporated into subsequent treatment decision-making.

Postoperative radiotherapy was routinely recommended for patients with residual disease following surgery and for patients who underwent surgery for recurrent tumors. In patients who achieved gross total resection without radiologically detectable residual disease, the potential benefits and risks of adjuvant radiotherapy were discussed individually. Adjuvant radiotherapy was generally favored in patients considered to have a higher risk of recurrence based on clinicopathological and surgical factors. The final treatment decision was made jointly by the treating physicians and the patient through a shared decision-making process.

Toxicity data were retrospectively extracted from follow-up records. Acute toxicities were defined as adverse events occurring during radiotherapy or within 90 days after treatment completion, whereas toxicities occurring thereafter were classified as late toxicities.

### 2.4. Target Delineation and Radiotherapy Planning

All patients underwent contrast-enhanced planning computed tomography (CT) and magnetic resonance imaging (MRI) before radiotherapy. CT and MRI datasets were fused for target delineation.

For conventionally fractionated radiotherapy (CFRT), the gross tumor volume (GTV) was defined as the residual contrast-enhancing tumor identified on postoperative MRI. In patients without visible residual disease, the postoperative surgical cavity and tumor bed were incorporated into the target volume. Areas considered at risk for microscopic tumor extension, including involved dura and radiologically suspicious dural tail when present, were included at the discretion of the treating radiation oncologist. A clinical target volume (CTV) margin of approximately 5–10 mm was applied and modified according to anatomical barriers and adjacent organs at risk. Subsequently, a planning target volume (PTV) margin of 3–5 mm was added to account for setup uncertainties and patient motion.

For stereotactic radiotherapy (SRT), the target volume generally consisted of the contrast-enhancing residual or recurrent tumor visualized on MRI. No additional CTV margin was routinely applied. A limited PTV margin of 1–3 mm was used according to institutional stereotactic radiotherapy protocols and image-guidance capabilities.

Radiotherapy was delivered using intensity-modulated radiotherapy (IMRT), helical IMRT, volumetric modulated arc therapy (VMAT), three-dimensional conformal radiotherapy (3D-CRT), or stereotactic radiotherapy (SRT). Daily image guidance was used according to institutional protocols to ensure treatment accuracy.

Conventionally fractionated radiotherapy was prescribed to a total dose of 50.4–60 Gy delivered in daily fractions of 1.8–2.0 Gy, five days per week. Patients with postoperative residual disease or recurrent tumors generally received higher total doses within this range. Conventionally fractionated radiotherapy was predominantly used in the postoperative adjuvant setting, whereas stereotactic radiotherapy was preferentially used in selected patients with recurrent disease or limited residual tumor volume. Stereotactic radiotherapy was delivered to a total dose of 25–30 Gy in 5 fractions. Treatment plans were normalized to ensure that at least 95% of the planning target volume received the prescribed dose, with prescription isodose lines typically ranging from 80% to 95%.

A summary of the treatment characteristics is presented in Table 2.

### 2.5. Follow-Up and Outcome Assessment

Patients were evaluated clinically and radiologically at regular intervals following treatment. Follow-up magnetic resonance imaging (MRI) was generally performed every 3 months during the first 2 years after radiotherapy, every 6 months between years 2 and 5, and annually thereafter.

Treatment response was assessed using serial follow-up MRI examinations in patients with measurable residual disease identified on the pre-radiotherapy MRI and was classified as complete response (CR), partial response (PR), stable disease (SD), or progressive disease (PD) according to radiological reports and multidisciplinary evaluation. Patients without measurable postoperative residual disease were excluded because no target lesion was available for radiological response assessment. Because of the retrospective nature of the study and the long study period, treatment response was determined based on radiological reports and multidisciplinary consensus rather than a prospectively defined response assessment protocol. Complete response (CR) was defined as the complete disappearance of measurable residual enhancing tumor on follow-up MRI. Partial response (PR) was defined as a reduction in measurable residual tumor burden, stable disease (SD) was defined as the absence of significant radiological change, and progressive disease (PD) was defined as radiological evidence of progression of residual disease, local recurrence, or the appearance of new tumor lesions.

Overall survival (OS) was defined as the time from the start of radiotherapy to death from any cause or last clinical follow-up. Local control (LC) was defined as the time from the start of radiotherapy to radiological evidence of recurrence or progression within the irradiated field. Patients without a local failure event were censored at the date of their last radiological assessment.

### 2.6. Statistical Analysis

Statistical analyses were performed using SPSS version 24.0 (IBM Corp., Armonk, NY, USA).

Categorical variables were summarized as frequencies and percentages, whereas continuous variables were reported as medians and ranges. Survival outcomes were estimated using the Kaplan–Meier method. Follow-up duration was estimated using the reverse Kaplan–Meier method. Number-at-risk tables were included for all Kaplan–Meier survival curves.

Univariate Cox proportional hazards regression analyses were performed to identify prognostic factors associated with OS and PFS. Variables with a *p*-value < 0.10 in univariate analyses were entered into multivariate Cox proportional hazards models. Hazard ratios (HRs) and corresponding 95% confidence intervals (CIs) were calculated. The proportional hazards assumption was evaluated prior to multivariable Cox regression analyses and no major violations were identified.

Because only eight local recurrence events were observed, multivariate Cox regression analysis for LC was not considered statistically appropriate. Given the limited number of outcome events, multivariable analyses should be considered exploratory and hypothesis-generating. A two-sided *p*-value < 0.05 was considered statistically significant.

## 3. Results

### 3.1. Patient and Treatment Characteristics

A total of 50 patients with histologically confirmed WHO grade 2 meningioma who underwent surgical resection followed by radiotherapy were included in this study. Baseline patient and tumor characteristics are summarized in Table 1, while treatment characteristics are presented in Table 2.

The median age was 56 years (range, 23–82 years). Twenty-three patients (46.0%) were female and 27 (54.0%) were male. Postoperative residual disease was present in 31 patients (62.0%).

The median interval between surgery and radiotherapy was 68.5 days (range, 21–2131 days). Most patients received adjuvant radiotherapy following primary surgery (n = 39, 78.0%). Six patients (12.0%) received adjuvant radiotherapy after surgery for recurrent disease, and five patients (10.0%) received salvage radiotherapy for recurrent tumors.

All patients received conventionally fractionated radiotherapy. VMAT was the most frequently used radiotherapy technique (n = 36, 72.0%), followed by IMRT (n = 11, 22.0%), helical IMRT (n = 2, 4.0%), and three-dimensional conformal radiotherapy (n = 1, 2.0%). Twenty-five patients (50.0%) received 54–56 Gy, while 25 patients (50.0%) received 60 Gy. Chemotherapy was administered to one patient (2.0%).

### 3.2. Treatment Response and Side Effects

Radiological response assessment demonstrated complete response in 15 patients (30.0%), partial response in 12 patients (24.0%), stable disease in 20 patients (40.0%), and progressive disease in three patients (6.0%).

Acute treatment-related adverse events were limited to Grade 1–2 toxicities. Radiation dermatitis was observed in four patients (8.0%), and dry eye was reported in one patient (2.0%). No Grade ≥3 acute adverse events were observed.

Late toxicity was limited to alopecia in one patient (2.0%). No Grade ≥3 late adverse events were recorded.

### 3.3. Survival Outcomes

Using the reverse Kaplan–Meier method, the median follow-up duration was 20.9 months (95% CI, 13.2–34.8 months). During follow-up, 10 patients (20.0%) died and seven patients (14.0%) developed local recurrence.

**Figure 1:**
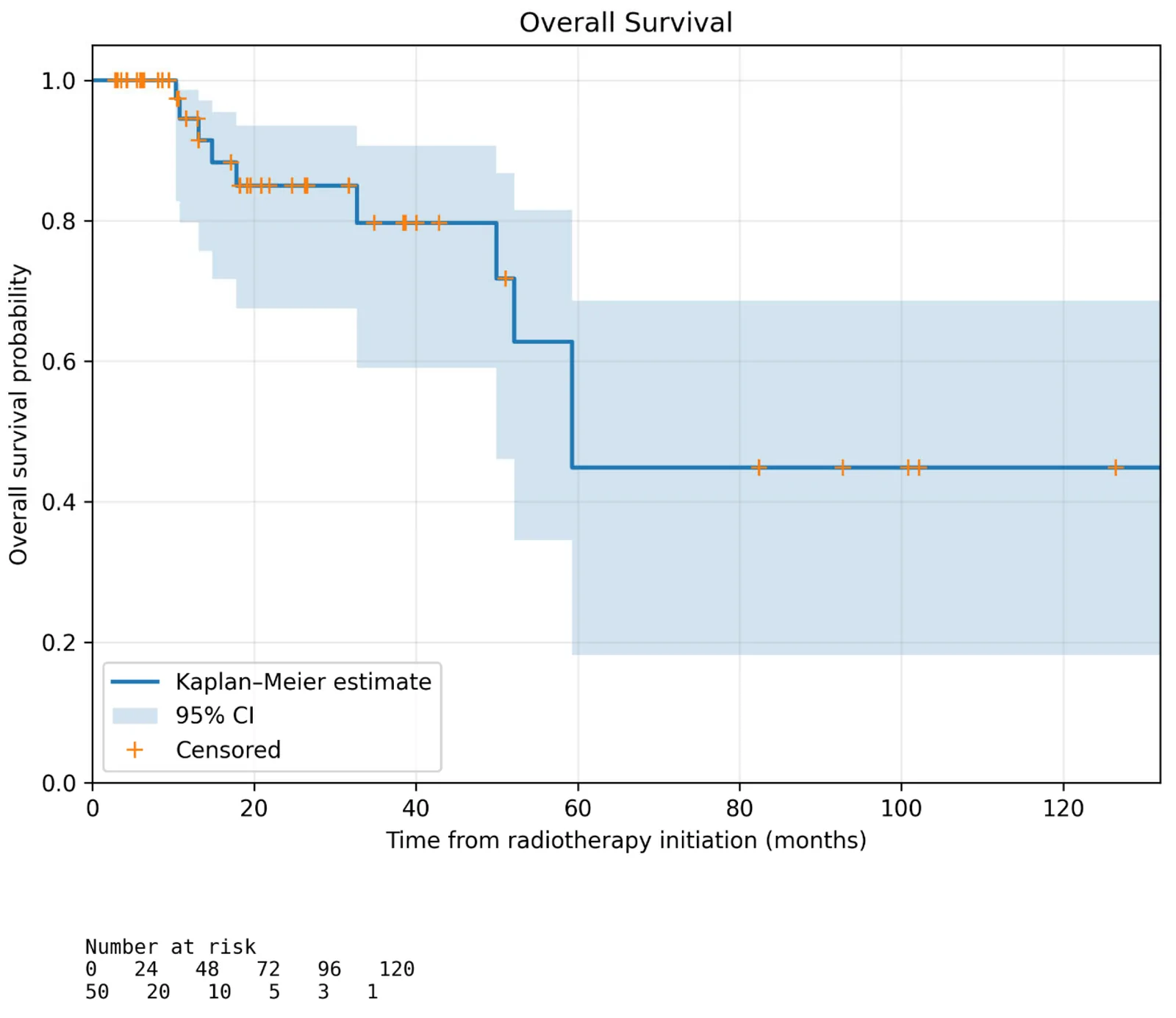
Kaplan–Meier curve for overall survival (OS).

The median overall survival was 59.3 months (95% CI, 49.9 months—not estimable). The estimated 1-, 2-, and 5-year overall survival rates were 94.5% (95% CI, 79.7–98.6%), 85.0% (95% CI, 67.6–93.5%), and 44.8% (95% CI, 18.1–68.6%), respectively (Figure 1).

**Figure 2:**
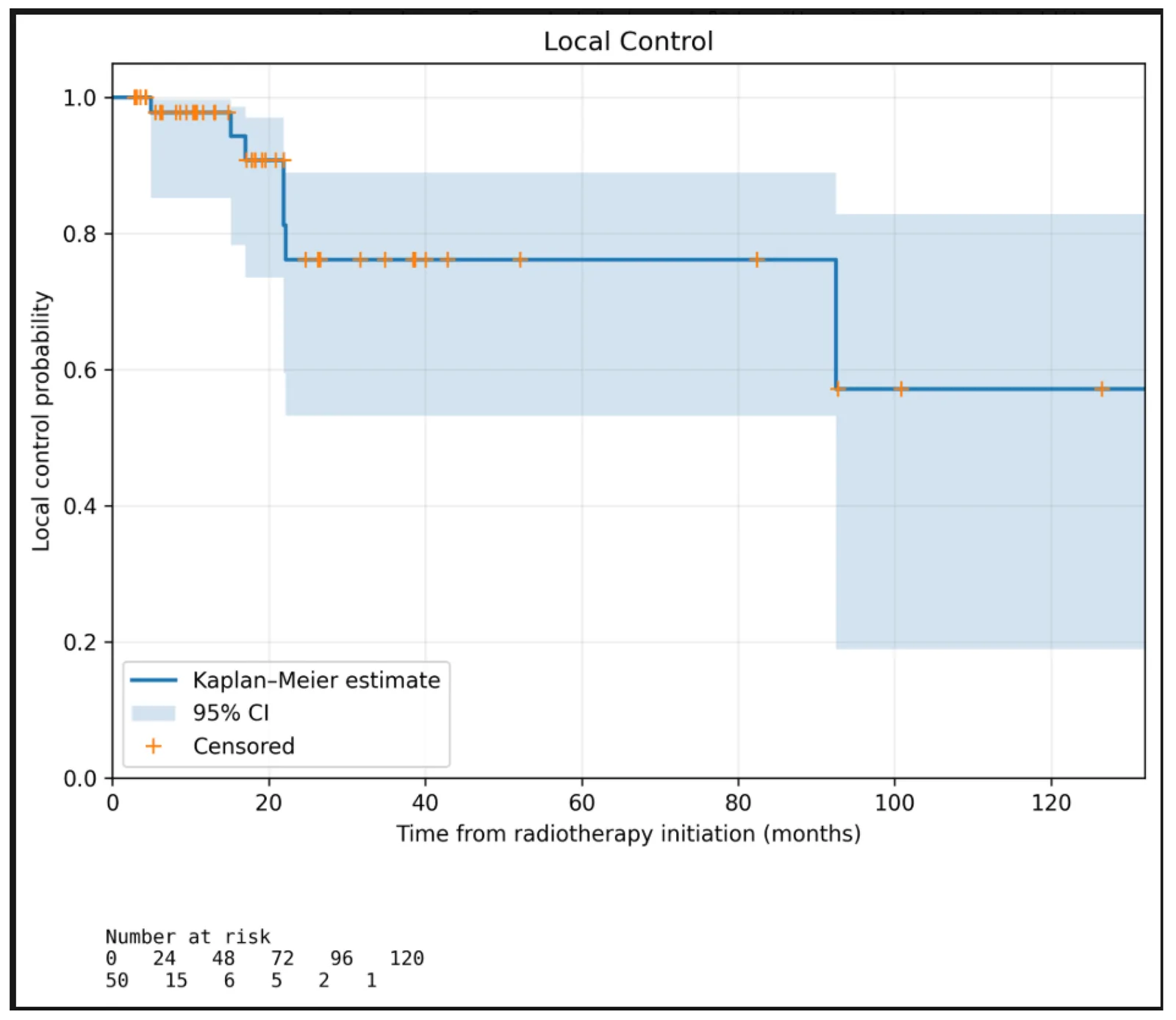
Kaplan–Meier curve for local control.

Median local control was not reached. The estimated 1-, 2-, and 5-year local control rates were 97.8% (95% CI, 85.3–99.7%), 76.2% (95% CI, 53.2–88.9%), and 76.2% (95% CI, 53.2–88.9%), respectively (Figure 2).

Figure 1 and Figure 2 present the Kaplan–Meier curves for overall survival and local control, including 95% confidence intervals, censoring marks, and number-at-risk tables.

### 3.4. Residual Disease Analysis

Postoperative residual disease was present in 31 patients (62.0%) and absent in 19 patients (38.0%). Clinical outcomes according to postoperative residual disease status are summarized in Table 3.

All deaths (n = 10) and all local recurrences (n = 7) occurred among patients with postoperative residual disease. No deaths or local recurrences were observed among patients without postoperative residual disease during follow-up.

Postoperative residual disease was significantly associated with death (Fisher’s exact test, *p* = 0.008) and local recurrence (Fisher’s exact test, *p* = 0.035). Because no outcome events occurred among patients without postoperative residual disease, the corresponding hazard ratios could not be reliably estimated using conventional Cox proportional hazards regression.

### 3.5. Prognostic Factors

The results of the univariable and multivariable Cox proportional hazards regression analyses for overall survival are summarized in Table 4 and Table 5, respectively. The results of the univariable Cox regression analyses for local control are presented in Table 6.

In the univariable analysis for overall survival, increasing age was significantly associated with poorer survival, with an HR of 3.47 for each 10-year increase in age (95% CI, 1.51–7.95; *p* = 0.003). ECOG performance status ≥1 was also significantly associated with poorer overall survival (HR, 8.64; 95% CI, 1.07–69.81; *p* = 0.043). Increasing tumor size was associated with poorer overall survival (HR per 1-cm increase, 1.68; 95% CI, 1.04–2.69; *p* = 0.033).

In the multivariable analysis, increasing age and tumor size remained independently associated with poorer overall survival. Each 10-year increase in age was associated with an adjusted HR of 2.41 (95% CI, 1.11–5.24; *p* = 0.026), while each 1-cm increase in tumor size was associated with an adjusted HR of 1.69 (95% CI, 1.08–2.64; *p* = 0.022). ECOG performance status ≥1 did not retain statistical significance after adjustment (adjusted HR, 6.58; 95% CI, 0.65–66.48; *p* = 0.110).

No evidence of violation of the proportional hazards assumption was identified for age, ECOG performance status, or tumor size in the multivariable overall survival model (all *p* > 0.05). Nevertheless, because only 10 deaths occurred, the adjusted estimates should be interpreted cautiously.

In the univariable analysis for local control, patients who had undergone more than one surgical procedure had significantly poorer local control than those who had undergone one surgical procedure (HR, 9.11; 95% CI, 1.76–47.17; *p* = 0.008). Treatment in the recurrent or salvage setting was also associated with poorer local control compared with primary adjuvant radiotherapy (HR, 8.64; 95% CI, 1.67–44.64; *p* = 0.010).

All local recurrences occurred among patients with postoperative residual disease. No local recurrence events occurred among male patients or patients with ECOG performance status 0. Consequently, hazard ratios for sex, ECOG performance status, and postoperative residual disease could not be reliably estimated because of complete separation. Because only seven local recurrence events were observed, a multivariable Cox regression model for local control was not constructed.

## 4. Discussion

The management of atypical meningioma (WHO grade 2) remains challenging despite advances in surgery, radiotherapy, and pathological classification. In the present study, we evaluated clinical outcomes in a single-center cohort of 50 patients with histologically confirmed WHO grade 2 meningioma who received radiotherapy following surgical resection. The estimated 2- and 5-year overall survival (OS) rates were 85.0% and 44.8%, respectively. Local control (LC) remained acceptable, with estimated 2- and 5-year LC rates of 76.2%. ECOG performance status and tumor size were identified as independent prognostic factors for OS.

Current evidence supports consideration of postoperative radiotherapy in patients with subtotal resection, postoperative residual disease, recurrent tumors, or other high-risk features. Prospective phase II studies, including NRG Oncology RTOG 0539 and EORTC 22042-26042, demonstrated favorable disease-control outcomes following postoperative radiotherapy in intermediate- and high-risk meningioma populations [15,16]. Retrospective series and pooled analyses have also reported improved disease control following postoperative radiotherapy, although the magnitude of benefit after gross total resection remains a matter of ongoing debate [6,7,8,9,10]. Multiple systematic reviews and meta-analyses have demonstrated improved local control and progression-free survival with adjuvant radiotherapy, whereas evidence supporting a clear overall survival benefit remains less consistent [11,12,13,14]. Our findings provide additional real-world evidence regarding outcomes following postoperative and salvage radiotherapy in patients with atypical meningioma.

One of the most notable findings of the present study was the strong association between postoperative residual disease and adverse clinical outcomes. All 10 deaths and all 7 local recurrences occurred among patients with postoperative residual disease, whereas no deaths or local recurrences were observed in patients without residual disease during follow-up. Hazard-ratio estimates for postoperative residual disease could not be reliably calculated because no events occurred in the subgroup without residual disease, resulting in complete separation. Incomplete resection and postoperative residual disease have repeatedly been associated with an increased risk of recurrence and inferior disease control in atypical meningioma [19,20,21,22,24,25]. The concentration of all adverse outcomes within the residual-disease subgroup further emphasizes the importance of maximal safe resection and supports consideration of postoperative radiotherapy in patients with residual tumor. Nevertheless, the absence of events among patients without residual disease should be interpreted cautiously because of the limited number of events, the relatively small cohort, and the duration of follow-up.

ECOG performance status emerged as a clinically meaningful prognostic factor for overall survival. Although previous studies have primarily focused on pathological, surgical, and treatment-related variables, patient-related factors may also substantially influence outcomes. Poor performance status may reflect greater neurological impairment, reduced physiological reserve, a higher comorbidity burden, and a reduced ability to tolerate subsequent treatment or salvage therapy. ECOG performance status may therefore represent a simple and readily available variable that can contribute to individualized risk stratification. However, the relatively small number of deaths resulted in wide confidence intervals around the hazard-ratio estimates. Accordingly, although the observed association appears clinically relevant, the magnitude of the effect should be interpreted cautiously and requires validation in larger cohorts.

Tumor size was also independently associated with overall survival. Previous studies have identified several tumor-related and histopathological characteristics as potential prognostic factors in atypical meningioma [19,23,24]. Larger tumors may be associated with greater surgical complexity, proximity to critical neurovascular structures, and an increased likelihood of postoperative residual disease. The association between increasing tumor size and inferior survival in our cohort may therefore partly reflect a greater postoperative tumor burden and more complex underlying disease. Because tumor size and residual disease may also be interrelated, this finding should be interpreted cautiously in view of the limited number of outcome events.

Patients treated for recurrent disease demonstrated significantly worse local control than those treated in the postoperative adjuvant setting. This finding is consistent with previous evidence indicating that outcomes may be less favorable when radiotherapy is deferred until recurrence or progression [26]. Recurrent tumors may be more difficult to control because of prior surgical manipulation, more extensive disease, unfavorable anatomical relationships, or treatment selection factors. Although our findings suggest a potential advantage of earlier radiotherapy integration in appropriately selected high-risk patients, the present study did not include a non-radiotherapy control group and therefore cannot establish the comparative benefit of routine postoperative radiotherapy.

The reverse Kaplan–Meier median follow-up was 20.9 months, with a 95% confidence interval of 13.2–34.8 months. Although several patients had substantially longer observation periods extending beyond 10 years, the number of patients remaining at risk progressively decreased at later time points. The estimated median OS was 59.3 months, with an upper confidence limit that could not be estimated. Consequently, the 5-year OS and LC estimates should be interpreted cautiously because relatively few patients remained at risk at later follow-up intervals. The number-at-risk tables accompanying the Kaplan–Meier curves provide additional information regarding the declining precision of these long-term estimates.

Another important consideration is the limited number of outcome events. Only 10 deaths and 7 local recurrences were observed. Multivariable Cox regression analysis was therefore restricted to OS and included only a limited number of clinically relevant variables to reduce the risk of model overfitting. A multivariable Cox model for LC was not performed because the number of local recurrence events was insufficient for reliable estimation. The limited event count may have contributed to the wide confidence intervals and reduced stability of some effect estimates. The multivariable findings should therefore be regarded as exploratory and hypothesis-generating rather than definitive evidence of causality.

The toxicity profile observed in our cohort was favorable. Acute adverse events were uncommon and limited to Grade 1–2 toxicity, and only one late adverse event was documented. No Grade 3 or higher late toxicity was observed. These findings are broadly consistent with prospective radiotherapy series reporting acceptable tolerability with contemporary conformal techniques [15,16]. However, toxicity data were retrospectively extracted from medical records, and low-grade or transient adverse events may consequently have been underreported.

### Limitations

This study has several limitations. First, its retrospective single-center design may limit the generalizability of the findings. Second, although follow-up was estimated using the reverse Kaplan–Meier method, the median follow-up duration remained relatively short, and long-term survival estimates should therefore be interpreted with caution. Third, the limited number of outcome events restricted multivariable analyses and may have contributed to the wide confidence intervals observed for several hazard-ratio estimates. Fourth, important clinicopathological variables, including histological subtype within WHO grade 2 meningioma, pial invasion, tumor location (convexity versus skull base), and Simpson resection grade, were not consistently available and therefore could not be incorporated into the analyses. Finally, because the study included only patients treated with radiotherapy and lacked a surgically treated non-radiotherapy control group, the independent effect of postoperative radiotherapy on survival outcomes cannot be determined.

## 5. Conclusions

Favorable local control and acceptable toxicity were observed following postoperative radiotherapy in patients with WHO grade 2 meningioma. Postoperative residual disease was strongly associated with adverse outcomes, as all observed deaths and local recurrences occurred in patients with residual disease. ECOG performance status and larger tumor size were independently associated with worse overall survival. Because of the retrospective design, limited number of outcome events, and the absence of a surgically treated non-radiotherapy control group, the independent effect of postoperative radiotherapy on survival outcomes cannot be determined. Larger prospective multicenter studies with longer follow-up are warranted to validate these findings and further refine patient selection for postoperative radiotherapy.

## Figures and Tables

**Table 1:** Baseline patient and tumor characteristics (n = 50).

Parameter	n (%)
Age, years	
Median (range)	56 (23–82)
<65 years	36 (71.0)
≥65 years	14 (28.0)
Sex	
Female	23 (46.0)
Male	27 (54.0)
ECOG performance status	
0	19 (38.0)
1	27 (54.0)
2	4 (8.0)
Tumor characteristics	
Preoperative tumor size, cm, median (range)	4.5 (2.0–9.5)
Ki-67 index, %, median (range)	10 (2–60)
Progesterone receptor expression, %, median (range)	40 (0–95)
Number of tumors	
Single	41 (82.0)
Multiple	8 (16.0)
Unknown	1 (2.0)
Dural tail sign	
Present	16 (32.0)
Absent	15 (30.0)
Unknown	19 (38.0)
Postoperative residual disease	
Present	31 (62.0)
Absent	19 (38.0)

Abbreviation: ECOG, Eastern Cooperative Oncology Group.

**Table 2:** Treatment characteristics and treatment-related side effects (n = 50).

Parameter	n (%)
Radiotherapy indication	
Adjuvant after primary surgery	39 (78.0)
Adjuvant after surgery for recurrence	6 (12.0)
Salvage radiotherapy after recurrence	5 (10.0)
Number of surgical procedures	
1	40 (80.0)
2	6 (12.0)
≥3	4 (8.0)
Interval between surgery and radiotherapy, days	
Median (range)	68.5 (21–2131)
Radiotherapy dose	
54–56 Gy	25 (50.0)
60 Gy	25 (50.0)
Radiotherapy technique	
IMRT	11 (22.0)
Helical IMRT	2 (4.0)
3D conformal radiotherapy (3D-CRT)	1 (2.0)
VMAT	36 (72.0)
Chemotherapy	
No	49 (92.0)
Yes	1 (2.0)
Acute side effects	
None	46 (92.3)
Dry eye	1 (2.0) *
Radiation dermatitis	4 (8.0)
Grade 1	3 (6.0)
Grade 2	1 (2.0)
Grade ≥3	0
Late side effects	
None	49 (98.0)
Alopecia	1 (2.0)
Grade ≥3	0

Values are presented as n (%) unless otherwise specified. Abbreviations: 3D-CRT, three-dimensional conformal radiotherapy; IMRT, intensity-modulated radiotherapy; VMAT, volumetric-modulated arc therapy. *: one patient had both dry eye and dermatitis.

**Table 3:** Outcomes according to postoperative residual disease status.

Residual Disease Status	Deaths n (%)	Local Recurrences n (%)
Absent (n = 19)	0 (0)	0 (0)
Present (n = 31)	10 (32.3)	7 (22.6)
*p* value *	0.008	0.035

* Fisher’s exact test.

**Table 4:** Univariable Cox proportional hazards regression analysis for overall survival.

Variable	Patients/Events	HR	95% CI	*p* Value
Age, per 10-year increase	50/10	3.47	1.51–7.95	0.003
Female vs. male	50/10	0.52	0.13–2.00	0.338
ECOG performance status ≥1 vs. 0	50/10	8.64	1.07–69.81	0.043
Ki-67 index, per 5% increase	50/10	1.21	0.92–1.60	0.173
Tumor size, per 1-cm increase	50/10	1.68	1.04–2.69	0.033
Postoperative residual disease, present vs. absent	50/10	Not estimable *	—	—
Multiple vs. solitary tumors	49/10	2.10	0.53–8.30	0.291
More than one vs. one surgical procedure	50/10	1.63	0.45–5.93	0.462
Recurrent/salvage setting vs. primary adjuvant RT	50/10	0.97	0.24–3.91	0.971
RT dose ≥60 vs. <60 Gy	50/10	1.65	0.46–5.92	0.440

* The hazard ratio could not be reliably estimated because no deaths occurred among patients without postoperative residual disease, resulting in complete separation. Abbreviations: CI, confidence interval; ECOG, Eastern Cooperative Oncology Group; HR, hazard ratio; RT, radiotherapy.

**Table 5:** Multivariable Cox proportional hazards regression analysis for overall survival.

Variable	Adjusted HR	95% CI	*p* Value
Age, per 10-year increase	2.41	1.11–5.24	0.026
ECOG performance status ≥1 vs. 0	6.58	0.65–66.48	0.110
Tumor size, per 1-cm increase	1.69	1.08–2.64	0.022

Abbreviations: CI, confidence interval; ECOG, Eastern Cooperative Oncology Group; HR, hazard ratio.

**Table 6:** Univariable Cox proportional hazards regression analysis for local control.

Variable	Patients/Events	HR	95% CI	*p* Value
Age, per 10-year increase	50/7	1.75	0.85–3.58	0.128
Female vs. male	50/7	Not estimable *	—	—
ECOG performance status ≥1 vs. 0	50/7	Not estimable *	—	—
Ki-67 index, per 5% increase	50/7	1.02	0.65–1.62	0.918
Tumor size, per 1-cm increase	50/7	1.07	0.59–1.94	0.831
Postoperative residual disease, present vs. absent	50/7	Not estimable *	—	—
Multiple vs. solitary tumors	49/7	3.54	0.63–19.79	0.150
More than one vs. one surgical procedure	50/7	9.11	1.76–47.17	0.008
Recurrent/salvage setting vs. primary adjuvant RT	50/7	8.64	1.67–44.64	0.010
RT dose ≥60 vs. <60 Gy	50/7	2.60	0.47–14.29	0.272

* The hazard ratio could not be reliably estimated because no local recurrence events occurred in one of the comparison groups, resulting in complete separation. Abbreviations: CI, confidence interval; ECOG, Eastern Cooperative Oncology Group; HR, hazard ratio; RT, radiotherapy.

## Data Availability

The datasets generated and/or analyzed during the current study are available from the corresponding author on reasonable request.
